# Quetiapine augmentation of SRIs in treatment refractory obsessive-compulsive disorder: a double-blind, randomised, placebo-controlled study [ISRCTN83050762]

**DOI:** 10.1186/1471-244X-5-44

**Published:** 2005-11-30

**Authors:** Paul D Carey, Bavanisha Vythilingum, Soraya Seedat, Jacqueline E Muller, Michael van Ameringen, Dan J Stein

**Affiliations:** 1 MRC Research Unit on Anxiety Disorders, University of Stellenbosch, Cape Town, South Africa; 2 Department of Psychiatry and Behavioral Neurosciences, McMaster University, Hamilton, Ontario, Canada

The comment in the introduction of our article [1] suggesting that "quetiapine is the only available antipsychotic with significant 5-HT1D effects," is misleading. The affinity of quetiapine for this receptor is low and probably insignificant when compared with some other second generation antipsychotics [2].

## Pre-publication history

The pre-publication history for this paper can be accessed here:



## References

[B1] Carey PD, Vythilingum B, Seedat S, Muller JE, van AM, Stein DJ (2005). Quetiapine augmentation of SRIs in treatment refractory obsessive-compulsive disorder: a double-blind, randomised, placebo-controlled study [ISRCTN83050762]. BMC Psychiatry.

[B2] Richelson E, Souder T (2000). Binding of antipsychotic drugs to human brain receptors focus on newer generation compounds. Life Sci.

